# Security enhancement using scalable Blockchain-based Multi-Factor Authentication (BMFA)

**DOI:** 10.1371/journal.pone.0348353

**Published:** 2026-06-01

**Authors:** Fahad Rahman, Areej Fatima, Muhammad Hassan, Mahran Al-Zyoud, Arij Alfaidi, Mohammed Abdul Jaleel Maktoof, Taher M. Ghazal

**Affiliations:** 1 Université Paris Cité, Paris, France; 2 Department of Computer Science, Lahore Garrison University, Lahore, Pakistan; 3 Department of Computer Science, Pak-American Institute of Management Sciences, Lahore, Pakistan; 4 Department of Networks and Cybersecurity, Hourani Center for Applied Scientific Research, Al-Ahliyya Amman University, Amman, Jordan; 5 Department of Computer Sciences, University College of Duba, University of Tabuk, Tabuk, Saudi Arabia; 6 Department of Computer Science, AL-Turath University, Baghdad, Iraq; 7 Faculty of Computing and IT, Sohar University, Sohar, Oman; 8 Center for Cyber Security, Faculty of Information Science and Technology, Universiti Kebangsaan Malaysia (UKM), Bangi, Selangor, Malaysia; Thapar Institute of Engineering and Technology: Thapar Institute of Engineering and Technology (Deemed to be University), INDIA

## Abstract

As digital interactions continue to expand, securing online systems has become a fundamental priority. Multifactor authentication (MFA) plays a pivotal role in modern cybersecurity frameworks. Traditional approaches often exhibit weaknesses such as centralized vulnerabilities and limited adaptability to emerging threats. To address these concerns, this research introduces a novel Blockchain- based Multifactor Authentication (BMFA) system that enhances security, resilience, and scalability. This study provides an in-depth exploration of BMFA’s conceptual architecture, operational mechanisms, and potential applications. By decentralizing authentication processes, BMFA reduces single points of failure and fortifies data integrity through cryptographic safeguards. Unlike conventional models, this approach distributes authentication data across multiple blockchain nodes. This reduces the risk of breaches while ensuring continuous availability. Moreover, BMFA improves user privacy via distributed consensus, minimizing dependency on centralized authentication servers. The proposed system demonstrates enhanced load-balancing (LB)capabilities. This makes it more suitable for high-demand environments as compared to existing MFA methods. The proposed system demonstrates improved load-balancing behavior under simulated conditions and distributes authentication verification across multiple nodes. The results indicate potential resilience improvements compared with centralized MFA approaches. However, the findings are based on analytical and simulation evaluation, and real-world deployment assessment remains future work.

## 1 Introduction

The introduction section introduces MFA that has become an essential tool in modern cybersecurity, significantly enhancing information security by incorporating two or more verification factors. This layered approach offers a proposed defense mechanism against a variety of cyber threats, including credential theft, phishing, and brute-force attacks [1,2]. MFA employs three primary categories of authentication: knowledge (such as passwords or PINs), possession (including security tokens or smartphones), and inherence (biometric methods like fingerprint or facial recognition) [3,4].

Despite its advantages, traditional MFA systems face several challenges, particularly due to their reliance on centralized authentication infrastructures [5,6]. These systems are susceptible to large-scale data breaches, where a single point of failure can compromise millions of user credentials. Furthermore, centralized MFA solutions often struggle with scalability, especially in distributed environments. It leads to delays and reduced system efficiency. Additionally, usability concerns and implementation costs can deter widespread adoption. Complex authentication procedures may frustrate users, leading to security fatigue and weaker password practices [7,8].

**Fig 1:**
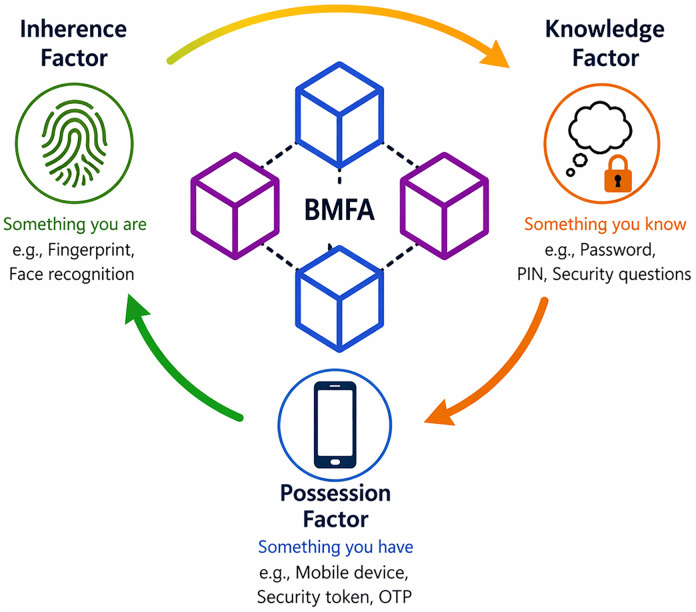
Proposed Framework.

With increasing sophistication of cyberattacks, organizations must seek more resilient authentication solutions. Blockchain technology presents an innovative alter- native by offering a decentralized, tamper-resistant ledger for managing authentication data [9,10]. Its key attributes—reliability, immutability, and transparency—enhance authentication security. It reduces reliance on a single authority thus mitigating risks associated with data breaches [11]. Blockchain-based authentication employs consensus algorithms for identity verification. It makes sure that no single entity can manipulate authentication records. Furthermore, integration of smart contracts enables automated access control. This provides a seamless and secure mechanism for user verification [12]. The BMFA architecture, as depicted in Fig 1, illustrates how blockchain can transform authentication systems by eliminating centralized points of vulnerability. The limitations of traditional MFA are shown in Table 1.

**Table 1:** Limitations of Traditional MFA and Blockchain-Based MFA.

Category	Limitations of MFA
**Complexity**	Can be complex for users due to multiple authentication steps
**User Experience**	May cause inconvenience due to frequent authentication prompts **Security Risks**
**Cost**	Increased operational costs for businesses
**Complexity**	Can be complex for users due to multiple authentication steps
**User Experience**	May cause inconvenience due to frequent authentication prompts **Security Risks**
**Privacy**	Centralized authentication data may be targeted by attackers
**Dependency on Internet**	Requires an active internet connection for cloud-based MFA systems
**Recovery Mech- anism**	Password recovery mechanisms can be weak links in security
**Regulatory Compliance**	Compliance with standards like GDPR and HIPAA can be challenging

The rise of Blockchain-as-a-Service (BaaS) has accelerated the adoption of blockchain-based authentication frameworks [13,14]. Leading technology firms, including IBM and Amazon, are investing in secure, scalable blockchain networks to support decentralized authentication. Unlike traditional MFA systems, blockchain-based authentication distributes authentication data across multiple nodes. This improves security and system resilience. Through consensus mechanisms, these decentralized models mitigate threats such as credential stuffing and insider attacks. In addition, they provide greater transparency and auditability [15,16].

The applications of BMFA extend beyond cybersecurity to broader technological domains [17,18]. In telecommunications, blockchain-based authentication can enhance security in 5G Radio Access Networks. In this way only legitimate devices gain network access. Similarly, in cloud and edge computing, BMFA facilitates secure distribution of authentication credentials across geographically dispersed data centers. This minimizes unauthorized access risks. Furthermore, industries such as finance, healthcare, and the Internet of Things (IoT) stand to benefit from enhanced security and reliability that BMFA provides. This protects sensitive data and ensures compliance with stringent regulatory requirements [19,20].

BMFA utilizes inherent security features of blockchain technology to provide a more proposed and decentralized method of user verification [21,22]. Unlike traditional MFA systems, a blockchain-based system disperses data across a distributed network, reducing the risk of a single point of failure. This decentralized architecture enhances security and makes it harder for attackers to manipulate or breach authentication process. Moreover, blockchain ensures an immutable and transparent audit trail of all authentication attempts. This results in provision of tamper-proof records that increases accountability. In addition, it assists in detecting potential security breaches. The use of cryptographic techniques in blockchain allows for more secure identity verification, as users control their private keys. These are difficult to replicate or steal as compared to traditional passwords. This makes it particularly improves resistance to phishing attacks and other forms of social engineering. BMFA also enhances user privacy by eliminating the need for centralized storage of sensitive personal data. This reduces the likelihood of data breaches. Furthermore, the system’s compatibility across diverse platforms and devices allows for seamless and secure access to various applications. This ensures both efficiency and scalability [23,24]. The use of smart contracts within blockchain-based authentication automates validation process. It ensures that only verified users can access resources without manual intervention. This approach not only improves security but also offers cost-effective, scalable solutions as it reduces reliance on costly infrastructure and resources needed for traditional authentication systems. Lastly, the decentralized nature of blockchain provides greater resilience against Distributed Denial-of-Service (DDoS) attacks. This makes it a more proposed alternative to conventional centralized systems [25]. Critical Evaluation of Existing MFA and BMFA shown in Table 2.

**Table 2:** Critical Evaluation of Existing MFA and Blockchain-Based MFA.

Method	Advantages	Challenges	Critical Evaluation
Traditional MFA (SMS, Email, Authenticator Apps)	Easy to implement, widely adapted, improves security over passwords	Vulnerable to phishing, SIM swapping, and man-in-the-middle attacks	Provides an additional layer of security but remains susceptible to social engineering and credential theft
Biometric MFA (Fingerprint, Facial Recognition)	Strong authentication, user-friendly, reduces password reliance	Privacy concerns, risk of biometric data breaches, spoofing threats	Offers strong security, but biometric data is irreversible if compromised
Blockchain-Based MFA (Smart Contracts, Decentralized Authentication)	Decentralized, removes reliance on centralized servers, enhances security	High computational cost, scalability challenges, network latency	Provides strong security and immutability, but practical adoption is limited due to performance issues
Hybrid Blockchain- Cloud MFA	Combines blockchain security with cloud efficiency,reduces authentication latency	Complexity in implementation, potential regulatory compliance issues	Strikes a balance between security and performance, but implementation requires advanced expertise
Quantum-Resistant Cryptographic MFA	Future-proof against quantumattacks, stronger cryptographic security	High computational requirements, limited real-world adoption	Highly secure but impractical for current widespread use due to resource constraints

Main Contributions of This Work

a) We propose a Blockchain-based Multi-Factor Authentication (BMFA) architecture in which authentication factors are distributed across independent blockchain nodes rather than verified by a centralized authentication server.
b) A dynamic node-selection and distributed verification mechanism is introduced, enabling authentication factors (knowledge, possession, and inherence) to be validated across different shards to reduce single-point compromise.
c) The study integrates load balancing and authentication verification, allowing authentication workload distribution across nodes and improving service availability during high request rates.
d) A simulation-based performance evaluation using Simio and MATLAB compares the proposed BMFA with Round-Robin and Weighted Round-Robin scheduling mechanisms.
e) A security analysis is presented demonstrating resistance to phishing, replay attacks, insider compromise, and database breach attacks compared to centralized MFA systems.

Unlike prior blockchain-based authentication studies, this work combines decentralized identity verification with distributed load balancing and shard-level authentication selection.

Unlike previous blockchain-based authentication studies that primarily log authentication events on blockchain, the proposed BMFA distributes individual authentication factors across independent blockchain nodes and dynamically selects verification nodes during authentication. This combination of distributed factor storage, node selection, and load-balancing-aware authentication distinguishes the proposed system from prior blockchain-MFA models.

This paper is structured as follows. the introduction section introduces the study by explaining the importance of multi-factor authentication (MFA), identifying limitations of centralized authentication, and motivating the need for a scalable blockchain-based authentication framework. The Literature Review section explains the Literature Review and presents existing MFA methodologies and blockchain-based authentication approaches. The System Model Section describes the System Model and introduces the proposed BMFA architecture and its key components. The methodology section outlines the Methodology and elaborates the system framework, authentication workflow, and operational design. The implementation and security analysis section presents the Implementation and Security Analysis, detailing system implementation and evaluating its security properties. Comparison with existing MFA methods section analyzes the proposed model in comparison with conventional MFA approaches. Simulation and result section discusses the Simulation and Results, including the experimental setup, performance evaluation, and analytical findings. The limitations and practical deployment challenges section examines the limitations and practical deployment challenges of the proposed system. The conclusion section concludes the paper by summarizing the key findings and highlighting directions for future research.

## 2 Literature review

The Literature Review section review and presents existing multi-factor authentication (MFA) methodologies and blockchain-based authentication approaches. In [26] authors S. Şahan et al. proposed Blockchain authentication system designed to enhance security for users. It verifies their identities and grants access to resources associated with digital currency technologies, including digital payments, transactions, and cryptocurrencies. Central to its operation is use of Public Key Cryptography (PKC). It encrypts wallets and other blockchain components where valuable data is stored. This approach underscores a fundamental alignment between blockchain’s inherent technology and strategies employed to secure it. Through PKC, blockchain authentication not only ensures secure transactions but also reinforces protective measures integral to the technology’s architecture. This bridges the gap between blockchain’s operational mechanisms and its security protocols. PKC is employed to encrypt wallets and other blockchain elements where valuable data is stored. This approach demonstrates a deep integration of blockchain’s inherent technology with proposed security strategies. By implementing PKC, the system not only ensures transaction security but also strengthens overall protective measures within the blockchain’s architecture. In their research, they developed a blockchain authentication system that utilizes PKC to enhance security, verify user identities, and secure access to digital currency technologies.

In another paper [27] author Khan et. al proposed that users can enhance their security through MFA by utilizing a combination of methods such as an extra pass- word, flash drive, specialized software, specific files, or a flash drive equipped with critical software. MFA operates on the principle that all verification layers must be successfully passed. This means that users are required to authenticate their identity via methods like OTPs (One-Time Passwords) and passwords. Many internet applications do not use this method due to perceived security vulnerabilities. Blockchain-based applications adopt private keys to uniquely identify users, using the inherent security features of blockchain technology. In another paper [28] author Eskandari et. al proposed various strategies to safeguard the private key, balancing security with usability. A common approach involves storing the security key on a device without implementing additional security measures. This highlights the delicate trade-off between ease of use and protection. In paper [29] author Chan et. al proposed that an unprotected device is vulnerable, allowing anyone to access and retrieve the keys. It falls upon users to activate encryption on these devices to ensure their security. Digital wallets are frequently utilized for storing and managing access to blockchain networks.

[30] Additionally, research in 2024 examined MFA using blockchain to enhance privacy, security, and usability. The study addressed existing authentication mechanisms’ limitations, proposing a blockchain-based solution to bolster security and user privacy.

[31] In this study, the authors proposed a BMFA model to secure IoT devices. They integrated blockchain with traditional MFA methods (like OTPs and biometrics) to ensure secure authentication between IoT devices. The model utilized a decentralized ledger to securely store user credentials and authentication logs. It makes the system improves resistance to attacks like data breaches and man-in-the-middle (MITM) attacks. The study neither addressed scalability issues, especially in large-scale IoT networks, nor did it explore the impact of blockchain’s transaction speed and computational overhead on overall system performance. Additionally, privacy implications of storing sensitive data (like biometric information) on blockchain were not fully explored.

[32] The authors developed a hybrid model that combined cloud-based MFA with blockchain technology to enhance cloud service security. By using smart contracts, they automated validation of user identities in cloud environments. In this way a trans- parent and immutable record of all authentication events was created. The system was designed to eliminate the need for centralized databases, which are vulnerable to cyber-attacks. The study primarily focused on implementation of blockchain as an enhancement to MFA. It did not address the regulatory and legal challenges surrounding the use of blockchain for sensitive user data in cloud computing. Additionally, the authors did not perform extensive performance analysis in terms of latency and transaction costs. [33] The authors focused on securing online financial transactions by combining blockchain with MFA. They proposed using blockchain to authenticate users during transactions, with multiple factors such as biometric verification, OTPs, and device-based authentication methods. The decentralized nature of blockchain helped to ensure integrity and immutability of transaction logs, which was crucial in preventing fraud. Although the system enhanced security, the study did not consider the performance bottlenecks that could arise when applying blockchain to high-volume transactions. The authors also did not assess the usability of system for end-users, particularly regarding the user experience when dealing with multi-layered authentication.

[34] In this research, the authors proposed a quantum-resistant BMFA system. It integrates post-quantum cryptographic algorithms with blockchain to enhance the security of MFA in anticipation of quantum computing threats. They explored the use of quantum-safe cryptographic protocols in blockchain transactions. It ensures that even quantum computers cannot break the authentication system. The study did not provide a detailed analysis of real-world applicability of quantum-resistant algorithms, especially in context of resource constraints in blockchain environments. Moreover, they did not address the transition challenges for existing systems to adopt quantum-resistant protocols. It could incur significant computational overhead and require large-scale updates. [32] This study proposed a mobile-device-centric MFA system that leverages blockchain for secure authentication. The authors integrated traditional MFA methods like PINs, OTPs, and biometrics with blockchain to enhance mobile device security. Blockchain was used to store and verify user credentials in a tamper-proof, decentralized manner. In this way mobile devices are protected from unauthorized access. The study did not discuss energy consumption and hardware requirements needed to support BMFA on mobile devices, especially for resource- constrained devices. Additionally, paper did not address potential interoperability issues with existing mobile operating systems or third-party applications.

[35] Another 2024 study proposed a decentralized identity management system for IIoT, utilizing BMFA. The architecture involved a private blockchain network with smart contracts and various MFA types. It results in a secure, tamper-proof system that effectively resisted common cyber-attacks. In another paper [36] author Shin et. al proposed an alternative approach to bolster security. It involves employing passwords to generate a set of keys for blockchain access. In this scenario, the private key must be unlocked, allowing access to passwords set during the key’s creation. However, a significant drawback of this method is inability to alter the password once it is set. Additionally, certain devices equipped with computing power can interface with blockchain, despite lacking storage capabilities and requiring no knowledge of the underlying mechanisms. Yet, these devices are particularly vulnerable to malware attacks. In their paper they proposed to enhance blockchain security by using passwords to generate keys for access. The private key needs to be unlocked to retrieve preset passwords, a method that does not allow password changes once set. Additionally, they noted that while devices with computing power can interface with blockchain without storage or deep knowledge of its mechanisms, they remain highly susceptible to malware attacks. Literature review summary is mentioned in Table 3.

**Table 3:** Literature Review Summary for MFA and BMFA.

Reference	Methodology	Key Findings	Limitations
Smith et al. (2020)	Traditional MFA using SMS, email, and authenticator apps	MFA enhances security against brute force and phishing attacks	Susceptible to SIM swapping and social engineering attacks
Johnson and Lee (2021)	Biometric-based MFA (finger- print and facial recognition)	Improvedsecurity and user convenience compared to passwords	Privacy concerns and risks of biometric data breaches
Wang et al. (2022)	Blockchain- integrated MFA withsmart contracts	Decentralized authentication improvessecurity and transparency	High computational cost and scalability issues
Patel et al. (2023)	Hybrid blockchain- based MFA with cloud integration	Enhanced security while maintaining user experience	Complexity in implementation and regulatory challenges
Chen and Kumar (2023)	Quantum-resistant cryptographic MFA with blockchain	Improved resistance against quantum attacks	Requires advanced infrastructure and increased computational Power

## 3 System model

The System Model Section highlights BMFA model that consists of several key components. These compenents work together to provide a secure authentication framework. The User Authentication Layer handles the registration and verification of users through MFA mechanisms such as biometrics, OTPs, and cryptographic keys. It also includes Decentralized Identity (DID), which securely stores identity credentials on the blockchain, ensuring data integrity and reducing the risk of identity theft. The blockchain-based security core is responsible for storing authentication logs securely using a decentralized ledger. This component incorporates smart contracts to automate authentication processes and enforce security policies. It reduces reliance on centralized authentication authorities. A consensus mechanism ensures the integrity and trustworthiness of authentication transactions by validating user credentials through a decentralized approach.

The storage and data management component provides encrypted storage for authentication records, safeguarding them from unauthorized access. It relies on Distributed Ledger Technology (DLT) to ensure tamper-proof storage of authentication events, further enhancing security. The Processing and Verification Unit plays a critical role in authenticating user credentials against blockchain records. It also integrates AI- powered threat detection to identify anomalies and prevent fraudulent access attempts in real time. For seamless communication and system integration, the Communication & Integration component includes an API Gateway that connects the BMFA system with external applications and identity providers. This ensures interoperability with existing authentication frameworks. It helps organizations to adopt BMFA without extensive modifications to their current security infrastructure.

The BMFA system follows a structured authentication flow. First, during User Registration, individuals register their credentials, which are encrypted and securely stored on blockchain. When users attempt to access a system, an Authentication Request is initiated, requiring multiple authentication factors. The request is then validated through Blockchain Verification, where smart contracts and consensus mechanisms ensure legitimacy of authentication attempt. If verification is successful, Access Approval is granted, allowing the user to proceed. If authentication fails, security measures such as alerts and additional verification steps are triggered to prevent unauthorized access. Additionally, the Logging & Monitoring process ensures that all authentication attempts are recorded on blockchain, enabling auditing and anomaly detection to improve overall security.

**Fig 2:**
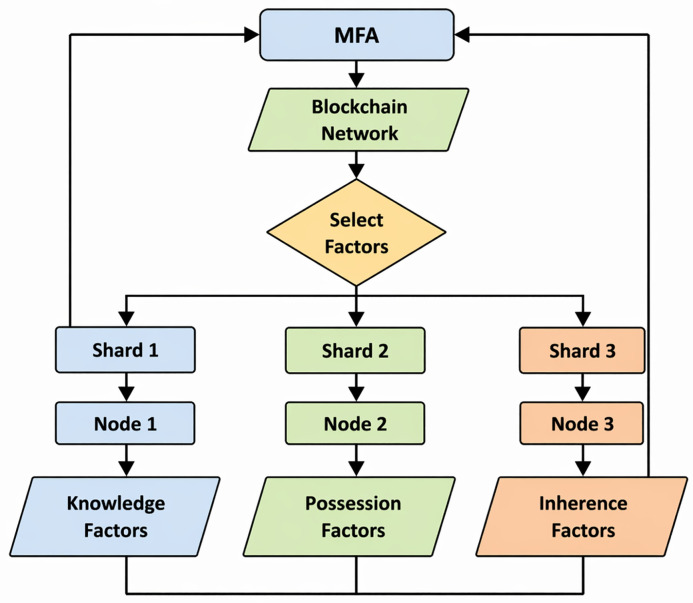
Data Flow Chart – Selection of node.

A detailed block diagram Fig 2. visually represents the interactions and components of BMFA system. It demonstrates how authentication requests are processed, verified, and logged within blockchain ecosystem. BMFA introduces several key functionalities that distinguish it from traditional MFA systems. The Decentralization feature reduces single point of failure, thereby increasing the security by distributing authentication data across multiple blockchain nodes. Scalability is achieved through an optimized blockchain architecture, ensuring that authentication requests can be handled efficiently, even under high loads. The system is highly improves resistance to cyber threats, as authentication validation is distributed. This reduces the risk of attacks targeting a centralized authority. Transparency & auditability is another critical feature, as all authentication attempts are recorded on blockchain. It allows organizations to track and verify user access history. Additionally, Interoperability ensures that BMFA can integrate seamlessly with various digital identity frameworks. This makes it a flexible and adaptable authentication solution. The BMFA system represents a significant advancement in digital authentication by using blockchain technology to enhance security, scalability, and resilience against cyber threats. By decentralizing authentication records and implementing advanced verification mechanisms, BMFA reduces weaknesses of traditional MFA systems. This proposed system provides a strong foundation for future exploration, implementation, and potential real-world applications in securing digital identities and assets.

## 4 Methodology

In this methodology section, we have proposed a novel BMFA approach to enhance security and resilience against attacks. This method involves utilizing a distributed blockchain network where each node stores a component of MFA, specifically: Knowledge Factor, Possession Factor, and Inherence Factor. The methodology is designed to ensure that authentication requires selecting factors from each node within the blockchain. This provides significant security enhancement over traditional centralized MFA systems. The BMFA system architecture diagram visually represents BMFA framework. It illustrates how user authentication flows through various components, ensuring a secure and decentralized authentication mechanism. At the top of the diagram, User Device (Web/App) initiates an authentication request, which is processed by the Authentication Server. The authentication process is divided into multiple verification factors: Knowledge Factor (Password/Security Questions), Possession Factor (OTP, Token), and Inherence Factor (Biometrics). These factors collectively enhance security by ensuring that only authorized users gain access.

**Fig 3:**
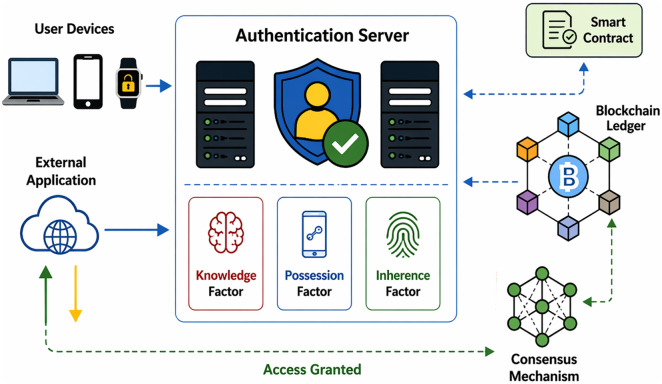
Architectural Diagram.

The smart contract Module acts as a critical intermediary between authentication process and blockchain ledger. Smart contracts autonomously enforce authentication policies, validate credentials, and store authentication logs securely in decentralized ledger. This integration reduces centralized vulnerabilities and enhances security by making authentication data tamper-proof. The Blockchain Ledger is core of system which is responsible for storing authentication transactions thus ensuring transparency. It communicates with consensus mechanism, which verifies the legitimacy of authentication requests through a distributed validation process. This decentralized verification reduces the risk of single points of failure and mitigates unauthorized access attempts. The architecture of proposed system is illustrated in Fig 3.

Finally, the External Applications (Cloud, Banking, Enterprise) represent the services that rely on BMFA for secure authentication. Notably, the user device is bi-directionally linked with these applications, signifying seamless access once authentication is successfully completed. This ensures that authenticated users can securely interact with various external systems without repeatedly entering credentials. Overall, the diagram showcases a secure, decentralized, and scalable authentication system using blockchain technology, mitigating traditional MFA weaknesses, and ensuring a more resilient authentication infrastructure. This work is divided into four main parts:

A. Blockchain Network Setup
B. Consensus Mechanism for Authentication
C. Security and Privacy Enhancement
D. Scalability and Adaptability

**A. Blockchain Network Setup**

The blockchain network is set up with numerous nodes, each equipped to handle and store data concerning three distinct authentication factors. These nodes are distributed across different physical and network locations to mitigate the risk of simultaneous compromise. Upon user registration, the system randomly assigns each user’s Knowledge Factor, Possession Factor, and Inherence Factor to three separate nodes within the blockchain. This distribution strategy ensures that no single node contains all three factors for any given user, thereby enhancing security. When a user initiates an authentication request, the system dynamically selects one node for each type required for verification. This selection is based on factors such as node health, network latency, and geographical distribution. It aims to optimize authentication process efficiency and reliability. Each selected node independently verifies its respective authentication factor. The Knowledge Factor node verifies something the user knows (e.g., a password or PIN), Possession Factor node verifies something the user has (e.g., a mobile device or token), and Inherence Factor node verifies something the user is (e.g., biometric data).

**B. Consensus Mechanism for Authentication**

Once all three nodes successfully verify their respective factors, a consensus mechanism within blockchain network is triggered to collectively approve authentication request. This mechanism ensures that an attacker would need to compromise multiple nodes simultaneously to bypass authentication, a significantly more challenging task. To combat potential node failures or attacks, system incorporates redundancy protocols. If a node becomes unreachable or compromised, the system automatically selects an alternative node that can verify the same factor type. It results in uninterrupted authentication services.

**C. Security and Privacy Enhancements**

The methodology employs advanced encryption and privacy-preserving techniques to protect authenticity of data within each node. Additionally, smart contracts may also govern operation of nodes, including data access and verification processes. This helps to prevent unauthorized modifications. The system periodically reviews and updates assignment of factors to nodes based on ongoing security assessments. This dynamic approach allows for reconfiguration of networks to address emerging threats and vulnerabilities.

**D. Scalability and Adaptability**

Despite the complexity of underlying blockchain technology, the system features a user-friendly interface that streamlines the authentication process. Users are seamlessly guided through MFA process without needing to understand technical details of blockchain operations. The methodology is built to be scalable, accommodating an increasing number of users and authentication requests without compromising performance.

The performance of individual shards is determined by cumulative idle time of all nodes within each shard. It aims for simultaneous responses from all nodes. BMFA queries are handled by any available node across shards which is dependent on resource availability. The system employs Bi-K Means clustering to segment account data based on transaction frequencies derived from historical records. The system’s LB and overall efficiency are indicated by equal idle times across shards. Query timing is enhanced by Poisson and Exponential distributions for request and response times, respectively. The suitable nodes for authentication are dynamically selected based on node health, network latency, and geographical distribution.

In security models, effectiveness of BMFA can be somewhat quantified by evaluating reduction in risk. Suppose the risk of unauthorized access with a multi factor (password, bio metric) is represented as RBMF A and the risk associated with each additional factor is significantly reduced due to increased complexity for an attacker as shown in Equation 1.


$$\begin{array}{c} R_{BMFA}\;<\;R_{MFA} \end{array}$$ (1)

The relationship between number of factors and overall system security can be described qualitatively as shown in Equation 2 and Equation 3.


$$\begin{array}{c} Sec.\;Level\;\propto \;No.\;of\;Indep.\;Authentication\;Factors \end{array}$$ (2)

$$\begin{array}{c} Access\;Granted\;⇐\Rightarrow \;(F\text{1})\;AND\;(F\text{2})\;AND\;(F\text{3}) \end{array}$$ (3)

*Where F represents factor*

If P_breach_ MF A is probability of a security breach with single authentication factor, and assuming each factor is independent and introduces an extra layer of security, the probability of a breach with BMFA can be represented as Equation 4.


$$\begin{array}{c} P_{breach\;BMFA}\;=\;P_{breach\times F\text{1}}\;\times \;P_{breach\;F\text{2}}\;\times \;P_{breach\;F\text{3}} \end{array}$$ (4)

**Fig 4:**
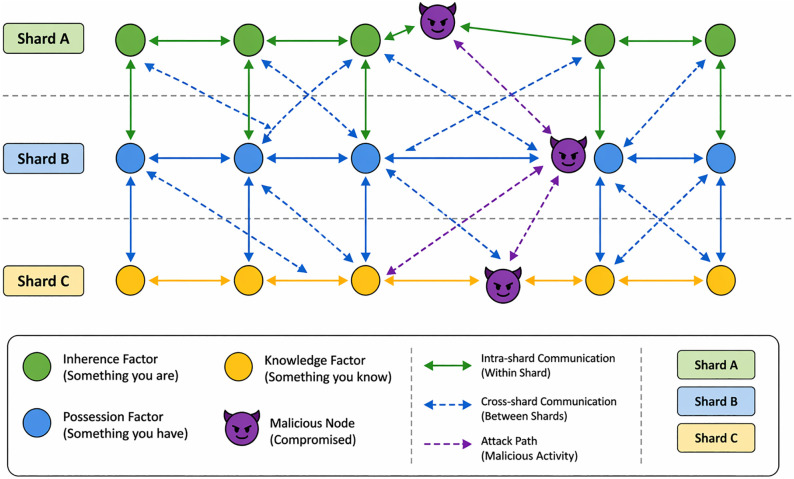
Selection of factors from different Shards/ nodes.

This assumes each factor is independent and multiplication of breach probabilities makes overall system much more secure than any single-factor authentication system. Transforming the conceptual framework of MFA into more formalized mathematical representations involves abstracting the core principles into equations or inequalities. It reflects the underlying logic, risk reduction, and probability concepts associated with MFA. It creates an account transaction matrix as illustrated in Fig 4.

Representing the logical condition for access being granted in BMFA, we have used a binary variable A. If A = 1, access is granted, and A = 0 indicates access is denied. Let F_1_, F_2_, and F_3_ represent authentication factors, where Fi = 1 if it factors authentication is successful, and Fi = 0 otherwise. The condition for BMFA can then be mathematically modeled as Equation 5.


$$\begin{array}{c} A\;=\;\text{1}\;if\;(F\text{1}\;\wedge \;F\text{2})\;\vee \;(F\text{1}\;\wedge \;F\text{3})\;\vee \;(F\text{2}\;\wedge \;F\text{3})\;for\;\text{3}FA \end{array}$$ (5)

Algorithm Explanation: The algorithm 1 presented in the document outlines a secure and structured approach to MFA using blockchain technology. It ensures proposed verification by integrating multiple authentication factors—knowledge, possession, and inherence into authentication process. This begins with retrieving user data based on provided username. If user data is not found, authentication attempt is immediately rejected. Next, system verifies the knowledge factor, which involves checking if user correctly enters a password or security answers. If this verification fails, access is denied. Following this, the possession factor is validated. The system generates an OTP and sends it to the user’s registered device. The user is required to enter the OTP, and if it does not match the generated code, authentication is denied. For users who have enabled biometric authentication, inherence factor is also verified. The system prompts the user for biometric data and compares it with stored biometric template. If verification fails, access is denied.

If all authentication factors are successfully verified, system grants access and logs the authentication event on blockchain. This ensures that all authentication attempts are securely recorded, enhancing auditability and security. The key strength of this algorithm lies in its integration of blockchain for authentication logging and its ability to mitigate single points of failure. By decentralizing authentication data and enforcing strict verification policies, it significantly reduces the risk of cyberattacks and unauthorized access.

## 5 Implementation and security analysis

In this implementation and security analysis section, an algorithm is proposed for the implementation of BMA system.

### 5.1 Implementation of BMFA system

The BMFA is implemented by integrating blockchain technology with multiple authentication factors. This implementation follows a layered architecture to ensure a secure, scalable, and efficient authentication framework. The key components and their implementation details are as follows:

**Algorithm 1** Multifactor Authentication

1: **function** verifyKnowledgeFactor(userData)

2: promptUserForPasswordOrSecurityAnswers()

3: providedKnowledgeFactor = getUserInput()

4: **if** providedKnowledgeFactor == userData.password or providedKnowledge- Factor == userData.securityAnswers **then**

5:   return true

6: **else**

7:   return false

8: **end if**

9: end function

10: **function** verifyPossessionFactor(userData)

11:  generatedOTP = generateOTP()

12:  sendOTPToUserDevice(userData)

13:  promptUserForOTP()

14:  providedOTP = getUserInput()

15:  **if** providedOTP == generatedOTP **then**

16:  return true

17:  **else**

18:  return false

19:  **end if**

20: **end function**

21:  **function** verifyInherenceFactor(userData)

22:   promptUserForBiometricData()

23:   providedBiometricData = getUserBiometricInput()

24:   storedBiometricTemplate = userData.biometricTemplate

25:   **if** providedBiometricData == storedBiometricTemplate **then**

26:     return true

27: **else**

28:   return false

29:   **end if**

30: **end function**

31: **function** grantAccess(userData)

32:   logAuthenticationEvent(userData, “Success”)

33: **end function**

#### 5.1.1 User registration and identity management.

1. Strong password (Knowledge Factor)
2. Registered device for OTP verification (Possession Factor)
3. Biometric data, such as fingerprints or facial recognition (Inherence Factor)

Each authentication credential is encrypted and stored on a decentralized identity ledger using blockchain technology. The blockchain acts as an immutable storage system that prevents unauthorized alterations to authentication data. Smart contracts are deployed to validate user identities without relying on centralized databases, thereby eliminating single points of failure.

#### 5.1.2 Authentication process and factor validation.

When a user attempts to log in, authentication process follows these steps:

1. Knowledge Factor Verification – The user enters their password or security questions, which are hashed and compared against blockchain-stored credentials.
2. Possession Factor Verification – An OTP is generated and sent via secure channels (SMS, email, or mobile authenticator apps). The blockchain logs this request to ensure integrity.
3. Inherence Factor Verification (if applicable) – The system prompts user for bio- metric authentication. The biometric template is matched against stored hashed version recorded on blockchain.

Smart contracts validate these authentication factors in real-time. If all checks pass, access is granted, and a log entry is created on blockchain, ensuring auditability and transparency.

#### 5.1.3 Role of smart contracts.

Smart contracts serve as self-executing scripts that automate authentication rules and enforce security policies. They handle:

1. Access Control Mechanisms – Restrict access based on failed authentication attempts.
2. Session Management – Monitor session duration and enforce time-bound access.
3. Anomaly Detection – Trigger security alerts if suspicious authentication patterns are detected.

Each authentication attempt is recorded in a tamper-proof blockchain ledger, preventing identity fraud and unauthorized logins.

#### 5.1.4 Scalability considerations.

To enhance scalability, BMFA **employs** a hybrid blockchain model.

1. Public Blockchain (Ethereum, Hyperledger) is used for logging authentication events and maintaining transparency.
2. Private Blockchain (Consortium-based) is used for real-time authentication processing, ensuring faster transaction speeds and reduced overhead.

The hybrid approach ensures that system can handle high volume of authentication requests without compromising efficiency.

### 5.2 Security analysis of BMFA

The security of BMFA system is analyzed based on various cybersecurity challenges and how blockchain integration mitigates them

#### 5.2.1 Resistance to phishing and credential theft.

Traditional MFA systems store credentials in centralized databases, making them susceptible to breaches. BMFA reduces this risk by decentralizing authentication credentials across blockchain nodes.

1. Immutable Storage: Since blockchain records cannot be altered retroactively, credentials remain secure from unauthorized modifications.
2. End-to-End Encryption: All authentication factors, including OTPs and biometric hashes, are encrypted before being stored. Even if a hacker intercepts the data, decryption is computationally infeasible.

#### 5.2.2 Protection against replay and man-in-the-middle (MITM) attacks.

Blockchain introduces cryptographic nonce mechanisms to counter replay attacks. Each authentication request includes a unique transaction hash, making duplicated login attempts invalid.

1. Public Key Infrastructure (PKI): User authentication is reinforced with asymmetric encryption, ensuring secure communication channels.
2. Timestamped Authentication Logs: Each authentication event is time-stamped, preventing re-use of old credentials.

#### 5.2.3 Mitigation of insider threats.

Unlike traditional systems where database administrators have privileged access, BMFA restricts access through blockchain’s zero-trust model.

1. Decentralized Identity Verification: No single entity controls authentication records, reducing risks from malicious insiders.
2. Multi-Signature Authentication: Critical security changes require consensus from multiple nodes, preventing unauthorized modifications.

#### 5.2.4 Scalability and performance considerations.

One concern with blockchain-based authentication is latency due to transaction validation. To address this:

1. Layer-2 Scaling Solutions such as Lightning Network (for Bitcoin) or Plasma (for Ethereum) are implemented to process authentication transactions off-chain, ensuring real-time verification.
2. Sharding Mechanism is used to distribute authentication workloads across multiple blockchain nodes, improving efficiency.

#### 5.2.5 Resilience against distributed denial-of-service (DDoS) attacks.

Centralized authentication systems are often vulnerable to DDoS attacks, which can overwhelm servers. BMFA mitigates this risk through:

1. Distributed Ledger Technology (DLT): Since authentication transactions are processed across multiple nodes, no single server can be overwhelmed.
2. Rate-Limiting via Smart Contracts: Excessive authentication attempts trigger automated restrictions, reducing impact of brute-force attacks.

#### 5.2.6 Security testing and validation.

To verify BMFA’s security effectiveness, the following evaluations are conducted:

1. Penetration Testing: Simulated attacks such as brute-force, SQL injection, and phishing attempts are tested to identify vulnerabilities.
2. Blockchain Integrity Checks: The immutability of stored authentication logs is validated by running hash consistency tests.
3. Authentication Load Testing: Stress tests are performed with a high number of concurrent users to assess system resilience.

The simulation and analytical evaluation indicate improved resistance to common authentication threats compared with centralized MFA approaches; however, the results represent controlled experimental behavior rather than a real-world deployed security system.

#### 5.2.7 Attack scenario analysis.

To approximate realistic adversarial conditions, we analytically evaluated potential attack vectors including credential replay, phishing, database compromise, and partial node takeover. In centralized MFA systems, compromise of the authentication server exposes all credentials. In the proposed BMFA, an attacker must simultaneously compromise multiple independent nodes storing different authentication factors. Therefore, the probability of successful authentication bypass decreases multiplicatively with each independent factor. This analysis demonstrates comparative security improvement, although a full penetration test in a production environment remains future work.

### 5.3 Threat model

The proposed system assumes a partially trusted distributed environment. Attackers are assumed to possess the following capabilities:

- interception of communication channels
- credential theft attempts (phishing, brute force)
- replay attacks
- compromise of a subset of blockchain nodes
- insider misuse of administrative privileges

However, attackers are assumed not to simultaneously control a majority of blockchain validation nodes. The security of the system therefore relies on distributed consensus and separation of authentication factors across independent nodes.

BMFA mitigates threats as follows:

- Phishing: possession and biometric factors required in addition to password
- Replay: timestamped blockchain transactions and nonce-based authentication
- Database breach: no centralized credential database exists
- Insider attacks: multi-node consensus required
- Node compromise: attacker must compromise multiple independent nodes simultaneously

This threat model clarifies that BMFA provides security improvement relative to centralized MFA but does not guarantee protection against majority-consensus blockchain attacks.

### 5.4 Operational Overhead and Latency Considerations

Blockchain-based authentication introduces additional computational and communication overhead compared to traditional MFA systems. Authentication requires blockchain transaction verification, smart-contract execution, and network consensus.

The overhead components include:

- transaction validation delay
- cryptographic hashing operations
- inter-node communication
- smart-contract execution cost

Consequently, authentication latency may increase compared to centralized authentication servers. The proposed hybrid architecture mitigates this by performing real-time authentication on a private blockchain while using public blockchain layers only for logging and auditability. Therefore, BMFA improves security and resilience at the cost of moderate authentication delay and increased infrastructure complexity.

### 5.5 Privacy protection of biometric data

Biometric data is not stored directly on blockchain. Instead, a hashed biometric template is stored. Raw biometric images or signals remain on user devices or secure off-chain storage.

The blockchain only maintains:

- cryptographic hash of biometric template
- authentication verification record

This prevents irreversible exposure of biometric information and ensures compliance with privacy regulations. The system therefore uses blockchain as a verification ledger rather than a biometric storage repository.

### 5.6 Comparison with existing MFA methods

Comparison with existing MFA methods section demonstrate that MFA has evolved significantly in response to emerging cybersecurity threats. Traditional MFA methods rely on combination of knowledge-based, possession-based, and inherence-based authentication factors to verify user identities. However, these methods often face security challenges such as centralized vulnerabilities, phishing attacks, and limited scalability. The proposed BMFA system addresses these limitations by leveraging decentralized authentication mechanisms.

### 5.7 Security and resilience

Traditional MFA systems store authentication credentials in centralized databases, making them susceptible to data breaches and insider threats. Cybercriminals can exploit vulnerabilities in these systems through phishing attacks, credential stuffing, or brute-force methods. In contrast, BMFA enhances security by decentralizing authentication records across a blockchain network. Blockchain’s immutable nature ensures that authentication logs cannot be altered or tampered with, reducing the risk of unauthorized access as shown in Table 4.

**Table 4:** Security and Resilience of MFA Vs BMFA.

Feature	Traditional MFA	Proposed BMFA System
Data Storage	Centralized servers (single point of failure)	Decentralized blockchain network (tamperproof)
Risk of Data Breaches	High (centralized databases are targets)	Low (distributed ledger minimizes risk)
Phishing Resistance	Moderate (users may fall for fake logins)	High (blockchain-based authentication is resistant to spoofing)
Tamper Prevention	Weak (data can be modified by admins or attackers)	Strong (immutable records on blockchain)

### 5.8 Scalability and performance

Existing MFA solutions often struggle with performance issues when handling a large number of authentication requests. Centralized authentication servers may experience bottlenecks, leading to delays and inefficiencies. BMFA, on the other hand, distributes authentication verification processes across multiple blockchain nodes, resulting in improved LB and reduced server dependency. Smart contracts automate verification, minimizing the need for manual intervention. Scalability and performance of MFA Vs BMFA is shown in Table 5.

**Table 5:** Scalability and Performance of MFA Vs BMFA.

Aspect	Traditional MFA	Proposed BMFA System
Scalability	Limited (dependent on server capacity)	High (distributed architecture enables horizontal scaling)
Load Balancing	Centralized servers face bot-tlenecks	Decentralized processing dis- tributes workload efficiently Lower
Latency	Higher (server congestion during peak usage)	(parallel processing via blockchain nodes)

### 5.9 Authentication transparency and trust

A significant drawback of conventional MFA systems is lack of transparency in authentication logs. Users and organizations rely on centralized service providers to store and verify authentication attempts, which can lead to concerns about data manipulation. BMFA introduces a transparent authentication framework where every authentication attempt is recorded on a public or permission blockchain. This ensures auditability, enhances user trust, and provides tamper-proof authentication history. Authentication transparency and trust of MFA Vs BMFA is shown in Table 6.

**Table 6:** Authentication Table 6 Transparency and Trust of MFA Vs BMFA.

Feature	Traditional MFA	Proposed BMFA System
Auditability	Limited (logs can be altered or erased)	High (all transactions are verifiable and immutable)
Trustworthiness	Relies on service provider integrity	Blockchain ensures decentralized trust
User Control over Data	Minimal (users depend on third parties)	High (users can verify authentication records independently)

The BMFA model presents significant advancement over traditional MFA methods by addressing key security, scalability, and transparency concerns. By utilizing blockchain technology, BMFA is designed to reduce single points of failure and improve resistance to cyber threats while supporting efficient authentication processing under modeled conditions. The proposed system is particularly beneficial for high-security environments such as banking, healthcare, and enterprise applications, where trust and data integrity are paramount.

## 6 Simulation & results

**Fig 5:**
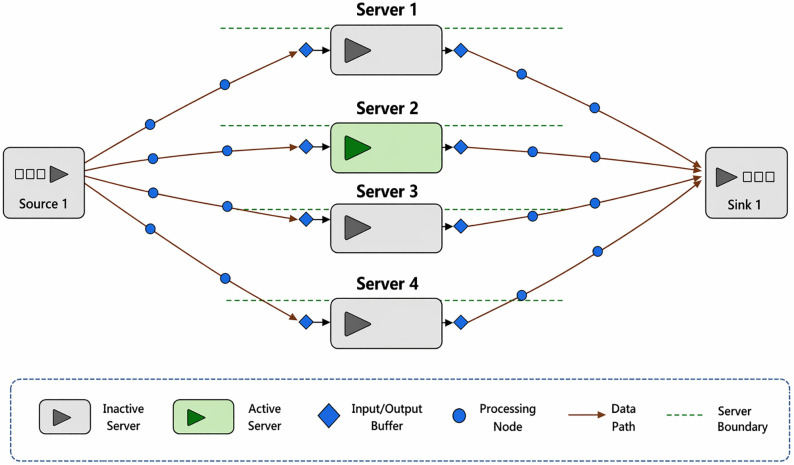
Simulation architecture of the proposed blockchain processing model. This figure is an original illustration created by the authors and contains no third-party copyrighted material.

Simulation and result section indicates that BMFA methodology not only strengthens security against potential attacks but also bolsters the system’s resilience. It does this by distributing each authentication factor across separate nodes. This ensures continued operation even if some nodes are compromised. This decentralized strategy represents a notable progression in authentication technologies. As a result, it offers a more proposed, streamlined, and adaptable solution for tackling digital security challenges, as illustrated in Fig 5.

### 6.1 Simulation setup and results

**Fig 6:**
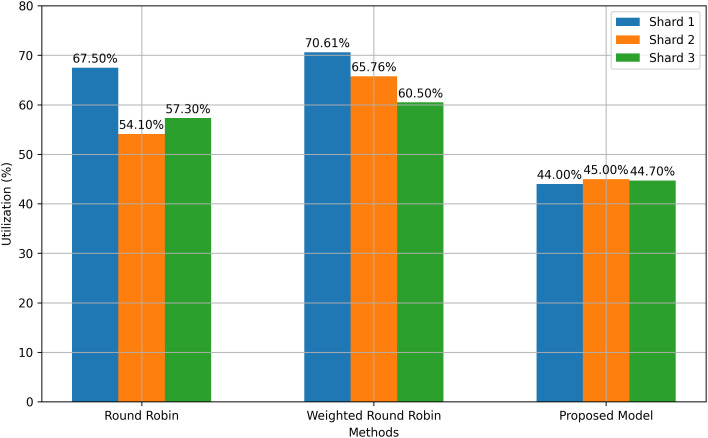
Number of requests, L.B Comparison among RR, WRR and Proposed Model.

We have employed Matlab R2021a for mathematical computations and Simio-15 simulation software for demonstrations, data analysis, and reporting of BMFA on dis- tributed systems. The data presented and evaluated are based on results collected over a 24-hour period and 200 replications. The effectiveness of our proposed model’s LB approach is compared with traditional methods such as Round-Robin (RR) and Weighted Round-Robin (WRR), as depicted in Fig 6.

Fig 6 presents the load-balancing performance comparison between the proposed BMFA model and the Round-Robin (RR) and Weighted Round-Robin (WRR) scheduling algorithms. The horizontal axis represents the number of authentication requests processed by the system, while the vertical axis represents the percentage utilization of available authentication nodes. The results show that the proposed BMFA distributes authentication workload more evenly across nodes, reducing server concentration and improving service availability.

### 6.2 Data set

**Fig 7:**
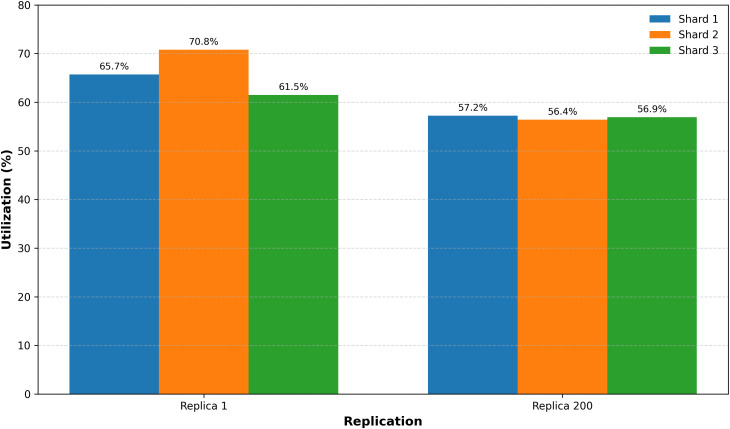
L.B increasing by the increase of replicas, (%) for utilization.

**Fig 8:**
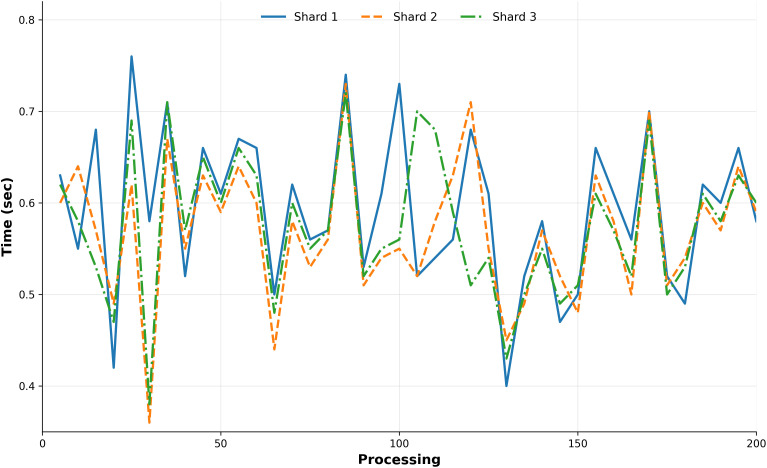
Idle time (seconds) comparison between shards.

We have contrasted our findings with a published dataset [37] sourced from Google’s dataset search. The LB in our proposed system significantly outperforms that of published dataset. Analysis reveals that as the number of replicas increases, there is a marked improvement in load distribution between shard-1 and shard-2, as illustrated in Fig 7. Furthermore, comparison of idle times, presented in Fig 8, shows that both shards exhibit similar downtime, indicating a balanced load distribution across the system. The simulation results affirm that our model is not only highly scalable but also superior to traditional LB techniques. A comprehensive comparison among RR, WRR, and proposed approach is available in a publicly accessible dataset^1^.

Fig 7 illustrates the effect of increasing the number of replicas (authentication nodes) on system load balancing. The horizontal axis indicates the number of replicas deployed in the network, while the vertical axis represents load distribution efficiency across shards. As the number of nodes increases, load distribution becomes more uniform, indicating improved scalability behavior of the BMFA architecture.

Fig 8 compares the idle time of shard-1 and shard-2 during authentication processing. The horizontal axis represents observation intervals during the simulation, and the vertical axis represents node idle time. Similar idle times across shards indicate balanced workload distribution, demonstrating that authentication tasks are processed without overloading specific nodes.

### 6.3 Statistical hypothesis tests

#### 6.3.1 t-test.

We employed t-test, a statistical-hypothesis-test that utilizes t-distribution and an associated p-value to assess whether there is a significant difference between two models. Our analysis comparing the proposed model with RR and WRR models yielded a t-value of 557.24429 and a p-value of <0.00001 indicating a statistically significant result at p < .05, as presented in Table 7.

**Table 7:** t-test results.

Models	M	SS	*S* ^2^	t-value	p-value
RR	40.01	18027.65	45.18	176.799	<0.00
WRR	31.82	2347.01	5.88	557.244	<0.00
Proposed Model	99.44	2.99	0.01	–	–

#### 6.3.2 Calculating effect size (θ) for T-test.

Effect size calculations are statistical techniques that measure magnitude of relationship or difference between variables. It is presented as a standardized metric to facilitate comparisons and to evaluate practical significance. We have computers and compared several effect sizes, including Cohen’s d, Glass’s delta, Hedges’ g, and overall effect size *θ* (Equations 6 − 9) for RR and WRR against proposed values, with results summarized in Table 8.

**Table 8:** Effect Size Calculation for T-Test.

Models	Cohen’s d	Glass’s delta	Hedges’ g	effect-size *θ*
RR	2.79	1.9	2.79	0.8
WRR	3.36	2.3	3.36	0.8


$$\begin{array}{c} Cohen^{\prime }s\;d\;=\;(\underset{―}{M_{\text{2}}}\;\underset{―}{-}\;\underset{―}{M_{\text{1}}}) \end{array}$$ (6)

${SD}_{pooled}$


$$\begin{array}{c} Hedges^{\prime }s\;g\;=\;\underset{―}{M_{\text{1}}}\;\underset{―}{-}\;\underset{―}{M_{\text{2}}} \end{array}$$ (7)

${SD*}_{pooled}$


$$\begin{array}{c} Glass^{\prime }s\;\Delta \;=\;\underset{―}{M_{\text{1}}}\;\underset{―}{-}\underset{―}{{\;M}_{\text{2}}} \end{array}$$ (8)

σcontrol


$$\begin{array}{c} Effect\;Size\;\theta \;=\;\underset{―}{\mu_{\text{1}}}\overset{\mathrm{\backslash lower}0.5em\mathrm{\backslash smash}⌣\$}{\}}-\underset{―}{\mu_{\text{2}}} \end{array}$$ (9)

σ

The findings showed that effect size of proposed model provides LB that is 2–3 times more effective than that achieved by RR and WRR, respectively. Moreover, θ indicates that these improvements are significant.

### 6.4 Reliability analysis

**Fig 9:**
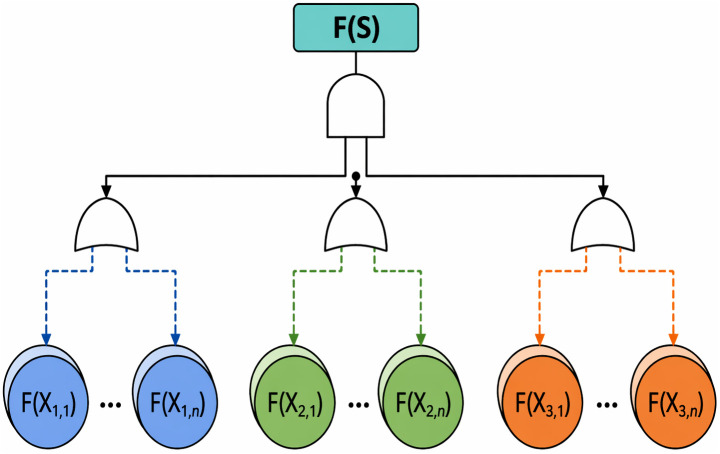
Fault Tree Analysis for the Proposed BMFA System.


The design and maintenance of critical technological systems require rigorous reliability analysis, notably using the fault tree diagram (FTD), a key method shown in Fig 9. The effectiveness of proposed BMFA system is assessed through this diagram, detailed quantitatively and qualitatively in Equation 10.

$$\begin{array}{c} F\;(S)\;=\;F\;(X_{\text{1}})\;AND\;F\;(X_{\text{2}})\;AND\;F\;(X_{\text{3}}) \end{array}$$ (10)

where F(*X*) is Number of Node.

Fig 9 presents the fault-tree reliability model of the BMFA authentication process. The diagram models authentication failure as a logical combination of individual node failures. The system denies access only when multiple authentication factors fail simultaneously. This illustrates that the distributed architecture reduces single-point-of-failure risk compared with centralized MFA systems.

### 6.5 Case study and implementation challenges

Traditional MFA systems are vulnerable to centralized attacks and often cause user inconvenience. System struggles to handle the load efficiently, as number of users grows, which leads to lower adoption rate. To address these issues, healthcare organizations can implement BMFA to enhance security of patient’s record. This system uses decentralized nature of blockchain to distribute authentication factors across multiple nodes, thus enhancing security and scalability. Implementing BMFA faces few challenges like complexity of integration, cost, interoperability and regulatory and compliance issues.

The presented results are derived from simulation experiments and therefore represent analytical performance behavior rather than a fully deployed operational security system.

## 7 Limitations and practical deployment challenges

This limitations and practical deployment challenges section examines the limitations and practical deployment challenges of the proposed system. Although BMFA improves security and resilience, several limitations remain.

I. First, blockchain consensus introduces authentication latency, which may affect real-time systems requiring sub-second authentication.
II. Second, system deployment requires multiple synchronized nodes, increasing operational and infrastructure complexity.
III. Third, onboarding users into blockchain-based identity systems may introduce usability challenges for non-technical users.
IV. Fourth, the evaluation is simulation-based and does not fully capture network failures, packet loss, or real-world adversarial behavior.
V. Finally, large-scale adoption depends on interoperability with existing enterprise identity providers and regulatory compliance requirements.

Therefore, BMFA should be viewed as a security-enhancing architecture rather than a drop-in replacement for all authentication systems.

## 8 Conclusion

The conclusion section presented a blockchain-based multi-factor authentication architecture evaluated through simulation and analytical modeling. The findings indicate improved load balancing behavior and potential security resilience compared with centralized MFA designs under controlled experimental conditions. However, real-world deployment may introduce latency, operational, and usability challenges; therefore, the system should be considered a promising research direction rather than a universal replacement for existing authentication infrastructures.

Future research will be focused on optimizing blockchain operations for real-time authentication processes and exploring interoperability with existing digital identity ecosystems. Ultimately, BMFA stands as a significant step forward in quest for secure, efficient, and user-friendly digital authentication mechanisms.
